# 4-Chloro-*N*-(pyrazin-2-yl)aniline

**DOI:** 10.1107/S1600536808041172

**Published:** 2008-12-13

**Authors:** Wan Ainna Mardhiah Wan Saffiee, Azila Idris, Zaharah Aiyub, Zanariah Abdullah, Seik Weng Ng

**Affiliations:** a Department of Chemistry, University of Malaya, 50603 Kuala Lumpur, Malaysia

## Abstract

In the title compound, C_10_H_8_ClN_3_, the dihedral angle between the aromatic rings is 43.0 (1)° and the bridging C—N—C angle is 128.19 (16)°. The amino N atom of one mol­ecule forms a hydrogen bond to the 1-N atom of an adjacent pyrazinyl ring, generating an inversion dimer.

## Related literature

For the two polymorphs of *N*-(pyrazin-2-yl)aniline, see: Wan Saffiee *et al.* (2008*a* ▶); Abdullah & Ng (2008 ▶). For *N*-(pyrazin-2-yl)-4-toluidine; see: Wan Saffiee *et al.* (2008*b* ▶).
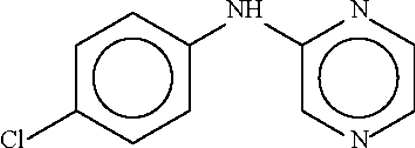


## Experimental

### 

#### Crystal data

C_10_H_8_ClN_3_
*M* *_r_* = 205.64
Monoclinic, 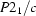

*a* = 12.1257 (3) Å
*b* = 3.7944 (1) Å
*c* = 19.7242 (5) Å
β = 91.370 (2)°
*V* = 907.25 (4) Å^3^
*Z* = 4
Mo *K*α radiation
μ = 0.38 mm^−1^
*T* = 100 (2) K
0.25 × 0.05 × 0.01 mm

#### Data collection

Bruker SMART APEX diffractometer
Absorption correction: multi-scan (*SADABS*; Sheldrick, 1996 ▶) *T*_min_ = 0.912, *T*_max_ = 0.996
7922 measured reflections
2073 independent reflections
1633 reflections with *I* > 2σ(*I*)
*R*_int_ = 0.033

#### Refinement

*R*[*F* ^2^ > 2σ(*F* ^2^)] = 0.037
*wR*(*F* ^2^) = 0.128
*S* = 1.14
2073 reflections
131 parameters
1 restraint
H atoms treated by a mixture of independent and constrained refinement
Δρ_max_ = 0.39 e Å^−3^
Δρ_min_ = −0.28 e Å^−3^

### 

Data collection: *APEX2* (Bruker, 2007 ▶); cell refinement: *SAINT* (Bruker, 2007 ▶); data reduction: *SAINT*; program(s) used to solve structure: *SHELXS97* (Sheldrick, 2008 ▶); program(s) used to refine structure: *SHELXL97* (Sheldrick, 2008 ▶); molecular graphics: *X-SEED* (Barbour, 2001 ▶); software used to prepare material for publication: *publCIF* (Westrip, 2009 ▶).

## Supplementary Material

Crystal structure: contains datablocks global, I. DOI: 10.1107/S1600536808041172/tk2340sup1.cif

Structure factors: contains datablocks I. DOI: 10.1107/S1600536808041172/tk2340Isup2.hkl

Additional supplementary materials:  crystallographic information; 3D view; checkCIF report

## Figures and Tables

**Table 1:** Hydrogen-bond geometry (Å, °)

*D*—H⋯*A*	*D*—H	H⋯*A*	*D*⋯*A*	*D*—H⋯*A*
N1—H1⋯N2^i^	0.88 (1)	2.15 (1)	3.023 (2)	171 (2)

Symmetry code: (i) 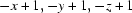.

**Fig. 1.**
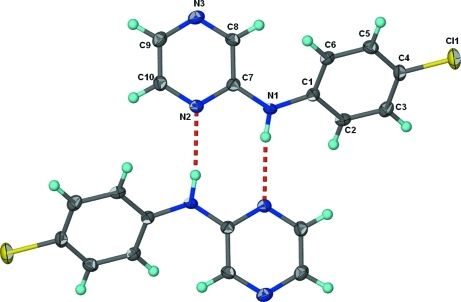
Thermal ellipsoid plot (Barbour, 2001) of hydrogen-bonded dimeric structure of C10H8ClN3 at the 70% probability level. Hydrogen atoms are drawn as spheres of arbitrary radius. Hydrogen bonds are shown as red dashed lines.

## supplementary crystallographic information

### Experimental

2-Chloropyrazine (1.15 g, 10 mmol) and 4-chloroaniline (1.28 g, 10 mmol) were mixed with ethanol (2 ml) and the mixture heated at 423–433 K for 3 h. The product was dissolved in water and the solution extracted with ether. The ether phase was dried over sodium sulfate; the evaporation of the solvent gave well shaped crystals along with some unidentified brown material.

### Refinement

Carbon-bound H-atoms were placed in calculated positions (C—H 0.95 Å) and were included in the refinement in the riding model approximation, with *U*(H) set to 1.2*U*_eq_(C).

The amino H-atom was located in a difference Fourier map, and was refined with a distance restraint of N–H 0.88±0.01 Å; its temperature factor was freely refined.

### Crystal data

**Table:**

C_10_H_8_ClN_3_	*F*(000) = 424
*M**_r_* = 205.64	*D*_x_ = 1.506 Mg m^−^^3^
Monoclinic, *P*2_1_/*c*	Mo *K*α radiation, λ = 0.71073 Å
Hall symbol: -P 2ybc	Cell parameters from 2160 reflections
*a* = 12.1257 (3) Å	θ = 2.6–28.1°
*b* = 3.7944 (1) Å	µ = 0.38 mm^−^^1^
*c* = 19.7242 (5) Å	*T* = 100 K
β = 91.370 (2)°	Plate, yellow
*V* = 907.25 (4) Å^3^	0.25 × 0.05 × 0.01 mm
*Z* = 4	

### Data collection

**Table:**

Bruker SMART APEX diffractometer	2073 independent reflections
Radiation source: fine-focus sealed tube	1633 reflections with *I* > 2σ(*I*)
graphite	*R*_int_ = 0.033
ω scans	θ_max_ = 27.5°, θ_min_ = 1.7°
Absorption correction: multi-scan (*SADABS*; Sheldrick, 1996)	*h* = −15→15
*T*_min_ = 0.912, *T*_max_ = 0.996	*k* = −4→4
7922 measured reflections	*l* = −25→24

### Refinement

**Table:**

Refinement on *F*^2^	Primary atom site location: structure-invariant direct methods
Least-squares matrix: full	Secondary atom site location: difference Fourier map
*R*[*F*^2^ > 2σ(*F*^2^)] = 0.037	Hydrogen site location: inferred from neighbouring sites
*wR*(*F*^2^) = 0.128	H atoms treated by a mixture of independent and constrained refinement
*S* = 1.14	*w* = 1/[σ^2^(*F*_o_^2^) + (0.0761*P*)^2^ + 0.0551*P*] where *P* = (*F*_o_^2^ + 2*F*_c_^2^)/3
2073 reflections	(Δ/σ)_max_ = 0.001
131 parameters	Δρ_max_ = 0.39 e Å^−^^3^
1 restraint	Δρ_min_ = −0.28 e Å^−^^3^

### Fractional atomic coordinates and isotropic or equivalent isotropic displacement parameters (Å^2^)

**Table:**

	*x*	*y*	*z*	*U*_iso_*/*U*_eq_	
Cl1	1.04801 (4)	−0.07942 (15)	0.36089 (3)	0.0269 (2)	
N1	0.59098 (13)	0.2944 (5)	0.43167 (8)	0.0173 (4)	
H1	0.5832 (19)	0.359 (6)	0.4742 (6)	0.019 (6)*	
N2	0.40848 (13)	0.4535 (5)	0.42294 (8)	0.0152 (4)	
N3	0.39085 (14)	0.1755 (5)	0.29204 (8)	0.0182 (4)	
C1	0.69716 (16)	0.1986 (5)	0.41131 (10)	0.0147 (4)	
C2	0.76584 (16)	0.0278 (5)	0.45861 (10)	0.0165 (4)	
H2	0.7381	−0.0315	0.5018	0.020*	
C3	0.87334 (16)	−0.0565 (5)	0.44384 (10)	0.0181 (4)	
H3	0.9196	−0.1710	0.4766	0.022*	
C4	0.91273 (16)	0.0283 (5)	0.38059 (11)	0.0173 (4)	
C5	0.84635 (16)	0.1980 (5)	0.33265 (10)	0.0176 (4)	
H5	0.8745	0.2545	0.2894	0.021*	
C6	0.73893 (16)	0.2849 (5)	0.34796 (10)	0.0155 (4)	
H6	0.6935	0.4033	0.3154	0.019*	
C7	0.49564 (16)	0.3053 (5)	0.39334 (9)	0.0141 (4)	
C8	0.48498 (16)	0.1632 (5)	0.32725 (10)	0.0159 (4)	
H8	0.5473	0.0556	0.3075	0.019*	
C9	0.30456 (17)	0.3280 (6)	0.32195 (10)	0.0185 (4)	
H9	0.2360	0.3451	0.2979	0.022*	
C10	0.31373 (16)	0.4595 (5)	0.38676 (10)	0.0168 (4)	
H10	0.2503	0.5588	0.4067	0.020*	

### Atomic displacement parameters (Å^2^)

**Table:**

	*U*^11^	*U*^22^	*U*^33^	*U*^12^	*U*^13^	*U*^23^
Cl1	0.0139 (3)	0.0324 (3)	0.0346 (3)	0.0038 (2)	0.0035 (2)	−0.0016 (2)
N1	0.0157 (8)	0.0266 (9)	0.0096 (8)	0.0025 (7)	0.0006 (6)	−0.0028 (7)
N2	0.0154 (8)	0.0179 (8)	0.0124 (8)	0.0017 (6)	0.0004 (6)	0.0011 (6)
N3	0.0190 (9)	0.0222 (9)	0.0134 (8)	−0.0051 (7)	−0.0009 (6)	0.0010 (7)
C1	0.0134 (9)	0.0158 (9)	0.0148 (9)	−0.0009 (7)	−0.0007 (7)	−0.0024 (7)
C2	0.0194 (10)	0.0181 (10)	0.0121 (9)	−0.0008 (8)	−0.0014 (7)	0.0000 (7)
C3	0.0180 (10)	0.0168 (10)	0.0192 (10)	0.0019 (8)	−0.0039 (8)	0.0009 (8)
C4	0.0121 (9)	0.0178 (10)	0.0219 (10)	−0.0001 (7)	0.0004 (7)	−0.0040 (8)
C5	0.0177 (10)	0.0203 (10)	0.0148 (9)	−0.0034 (8)	0.0028 (7)	−0.0018 (8)
C6	0.0155 (9)	0.0166 (9)	0.0142 (9)	−0.0008 (7)	−0.0017 (7)	0.0005 (7)
C7	0.0143 (9)	0.0154 (9)	0.0125 (9)	−0.0014 (7)	0.0003 (7)	0.0020 (7)
C8	0.0164 (10)	0.0186 (10)	0.0129 (9)	−0.0012 (8)	0.0024 (7)	−0.0008 (8)
C9	0.0154 (10)	0.0236 (10)	0.0163 (10)	−0.0025 (8)	−0.0018 (7)	0.0045 (8)
C10	0.0145 (9)	0.0192 (10)	0.0167 (10)	0.0008 (8)	0.0013 (7)	0.0026 (8)

### Geometric parameters (Å, °)

**Table:**

Cl1—C4	1.743 (2)	C3—C4	1.385 (3)
N1—C7	1.367 (2)	C3—H3	0.9500
N1—C1	1.406 (2)	C4—C5	1.386 (3)
N1—H1	0.881 (10)	C5—C6	1.384 (3)
N2—C10	1.338 (2)	C5—H5	0.9500
N2—C7	1.343 (2)	C6—H6	0.9500
N3—C8	1.323 (3)	C7—C8	1.414 (3)
N3—C9	1.344 (3)	C8—H8	0.9500
C1—C2	1.395 (3)	C9—C10	1.374 (3)
C1—C6	1.398 (3)	C9—H9	0.9500
C2—C3	1.380 (3)	C10—H10	0.9500
C2—H2	0.9500		
			
C7—N1—C1	128.19 (16)	C6—C5—H5	120.1
C7—N1—H1	114.2 (15)	C4—C5—H5	120.1
C1—N1—H1	117.6 (15)	C5—C6—C1	120.14 (18)
C10—N2—C7	116.74 (16)	C5—C6—H6	119.9
C8—N3—C9	117.11 (17)	C1—C6—H6	119.9
C2—C1—C6	118.89 (18)	N2—C7—N1	115.87 (17)
C2—C1—N1	117.73 (17)	N2—C7—C8	120.33 (17)
C6—C1—N1	123.26 (17)	N1—C7—C8	123.78 (18)
C3—C2—C1	121.21 (18)	N3—C8—C7	121.97 (18)
C3—C2—H2	119.4	N3—C8—H8	119.0
C1—C2—H2	119.4	C7—C8—H8	119.0
C2—C3—C4	118.96 (18)	N3—C9—C10	121.19 (19)
C2—C3—H3	120.5	N3—C9—H9	119.4
C4—C3—H3	120.5	C10—C9—H9	119.4
C3—C4—C5	121.05 (18)	N2—C10—C9	122.63 (18)
C3—C4—Cl1	119.53 (16)	N2—C10—H10	118.7
C5—C4—Cl1	119.42 (16)	C9—C10—H10	118.7
C6—C5—C4	119.75 (19)		
			
C7—N1—C1—C2	−146.4 (2)	N1—C1—C6—C5	176.58 (18)
C7—N1—C1—C6	37.6 (3)	C10—N2—C7—N1	−178.56 (17)
C6—C1—C2—C3	−0.1 (3)	C10—N2—C7—C8	−0.4 (3)
N1—C1—C2—C3	−176.21 (19)	C1—N1—C7—N2	−171.00 (19)
C1—C2—C3—C4	−0.5 (3)	C1—N1—C7—C8	10.9 (3)
C2—C3—C4—C5	0.6 (3)	C9—N3—C8—C7	−0.4 (3)
C2—C3—C4—Cl1	−179.50 (16)	N2—C7—C8—N3	1.2 (3)
C3—C4—C5—C6	0.0 (3)	N1—C7—C8—N3	179.22 (19)
Cl1—C4—C5—C6	−179.91 (15)	C8—N3—C9—C10	−1.1 (3)
C4—C5—C6—C1	−0.6 (3)	C7—N2—C10—C9	−1.1 (3)
C2—C1—C6—C5	0.7 (3)	N3—C9—C10—N2	1.9 (3)

### Hydrogen-bond geometry (Å, °)

**Table:**

*D*—H···*A*	*D*—H	H···*A*	*D*···*A*	*D*—H···*A*
N1—H1···N2^i^	0.88 (1)	2.15 (1)	3.023 (2)	171 (2)

Symmetry codes: (i) −*x*+1, −*y*+1, −*z*+1.
